# Monocrystalline diamond detector for online monitoring during synchrotron microbeam radiotherapy

**DOI:** 10.1107/S160057752300752X

**Published:** 2023-10-10

**Authors:** Francesca di Franco, Nicolas Rosuel, Laurent Gallin-Martel, Marie-Laure Gallin-Martel, Mostafa Ghafooryan-Sangchooli, Sarvenaz Keshmiri, Jean-François Motte, Jean-François Muraz, Paolo Pellicioli, Marie Ruat, Raphael Serduc, Camille Verry, Denis Dauvergne, Jean-François Adam

**Affiliations:** a Université Grenoble-Alpes, CNRS, Grenoble INP, LPSC UMR5821, 38000 Grenoble, France; b Université Grenoble-Alpes, UGA/INSERM UA7 STROBE, 2280 Rue de la Piscine, 38400 Saint-Martin d’Hères, France; c Université Grenoble-Alpes, Institut Néel, CNRS, Grenoble-INP, Grenoble, France; d ESRF, Grenoble, France; e Centre Hospitalier Universitaire Grenoble-Alpes, CS10217, 38043 Grenoble, France; Uppsala University, Sweden

**Keywords:** monocrystalline diamond detectors, synchrotron microbeam radiotherapy, online dosimetry

## Abstract

Microbeam radiation therapy (MRT) is a radiotherapy technique combining spatial fractionation of the dose distribution on a micrometric scale, X-rays in the 50–500 keV range and dose rates up to 16 × 10^3^ Gy s^−1^. Nowadays, *in vivo* dosimetry remains a challenge due to the ultra-high radiation fluxes involved and the need for high-spatial-resolution detectors. The aim here was to develop a striped diamond portal detector enabling online microbeam monitoring during synchrotron MRT treatments. The detector, a 550 µm bulk monocrystalline diamond, is an eight-strip device, of height 3 mm, width 178 µm and with 60 µm spaced strips, surrounded by a guard ring. An eight-channel ASIC circuit for charge integration and digitization has been designed and tested. Characterization tests were performed at the ID17 biomedical beamline of the European Synchrotron Radiation Facility (ESRF). The detector measured direct and attenuated microbeams as well as interbeam fluxes with a precision level of 1%. Tests on phantoms (RW3 and anthropomorphic head phantoms) were performed and compared with simulations. Synchrotron radiation measurements were performed on an RW3 phantom for strips facing a microbeam and for strips facing an interbeam area. A 2% difference between experiments and simulations was found. In more complex geometries, a preliminary study showed that the absolute differences between simulated and recorded transmitted beams were within 2%. Obtained results showed the feasibility of performing MRT portal monitoring using a microstriped diamond detector. Online dosimetric measurements are currently ongoing during clinical veterinary trials at ESRF, and the next 153-strip detector prototype, covering the entire irradiation field, is being finalized at our institution.

## Abstract 2

The development of a striped diamond portal detector for online monitoring during synchrotron microbeam radiation therapy is described. Its feasibility, ongoing dosimetric measurements in clinical trials and a prototype for a larger detector currently in progress are also described.

## 1. Introduction

Microbeam radiation therapy (MRT) is a radiotherapy technique developed at synchrotron radiation sources (Eling *et al.*, 2019 ▸) combining spatial fractionation of the dose distribution on a micrometric scale, X-rays in the 50–500 keV range and dose rates up to 16 × 10^3^ Gy s^−1^, enabling the so-called FLASH effect (Favaudon *et al.*, 2014 ▸; Eling *et al.*, 2019 ▸; Montay-Gruel *et al.*, 2022 ▸). Typical irradiation geometries are 25–50 µm-wide quasi-parallel beamlets (peak dose regions) equally spaced between 200 and 400 µm pitch, inducing so-called valley dose regions between the peaks. The peak dose deposited along the microbeams’ pathways can reach several hundred grays, whereas the valley doses (diffused between the microbeams) only make up 5–7% of the peak dose (Eling *et al.*, 2019 ▸). Recent preclinical studies demonstrated the potential of MRT to improve tumor control with fewer side effects (Smyth *et al.*, 2016 ▸; Fernandez-Palomo *et al.*, 2020 ▸; Eling *et al.*, 2021 ▸). Nowadays, synchrotron precision *in vivo* dosimetry poses a significant challenge due to the ultra-high-radiation fluxes involved, low-to-medium-energy photons, and the need for high-resolution detectors capable of resolving the dose distribution on a micrometric scale. Therefore, an online monitoring detector must have both general characteristics, such as adequate radiation hardness and a fast response time for real-time monitoring, and specific characteristics, such as high spatial resolution, an almost 100% charge collection efficiency, a wide dynamic range and an operational energy range of 50–500 keV, to accurately measure the deposited energy by the high photon flux in the detector. Furthermore, to link the measured dose to the actual delivered dose in the patient, the detector has to be tissue-equivalent.

Considering the above-mentioned properties, the detectors routinely used in conventional radiotherapy are not suitable for MRT. At present, several MRT detectors have been studied but none were found to be optimal (Bartzsch *et al.*, 2020 ▸). Silicon diodes and metal oxide semiconductor field-effect transistors (MOSFETs) exhibited a weak tissue-equivalence at MRT beam energies with responses severely dependent on the beam energy (Essers & Mijnheer, 1999 ▸; Parwaie *et al.*, 2018 ▸). On the other side, thermoluminescent detectors (TLDs), optically stimulated luminescence detectors (Spasic & Adam, 2013 ▸), radiochromic films (Ocadiz *et al.*, 2019 ▸) and gel dosimeters have shown a good tissue-equivalence in synchrotron radiotherapy beams but with different reading procedures, making their use impossible for online treatment monitoring. Moreover, the operational dose range of these detectors could be quite restrictive. For example, the TLDs signal exhibits a significant loss of linearity with dose when operating beyond 1 Gy (Mack *et al.*, 2002 ▸).

In the context of MRT, diamond detectors might be ideal candidates for online treatment monitoring owing to their physical properties. Diamond detectors are large-gap semiconductors (energy gap = 5.47 eV) operating as fast detectors under high radiation flux thanks to their high charge-carrier mobility (∼2000 cm^2^ V^−1^ s^−1^) (Pomorski *et al.*, 2006 ▸). Their atomic density compensates for their low atomic number (*Z* = 6, close to that of human tissues, *Z*_eff_ = 7.4), resulting in good detection efficiency. Moreover, diamonds have a high displacement threshold energy of 43 eV and high thermal conductivity, making them radiation-hard, a good detection efficiency for high-flux low-energy photons (keV range), a linear response with dose rate, and can perform real-time measurements. Additionally, a good spatial resolution may be achieved by means of precision-pixelated surface metallization. These characteristics suit well the requirements for an online monitoring device in MRT. The first commercialized monocrystalline diamond dosimeter was the PTW 60019 microdiamond (PTW, Freiburg, Germany), which was tested under a 220 kV photon beam (2 Gy min^−1^ dose rate) and showed good dose linearity, with a deviation of less than 0.5% for a dose between 1 and 20 Gy (Kampfer *et al.*, 2018 ▸). In addition, Livingstone *et al.* (2016 ▸) tested the PTW diamond dosimeter under synchrotron radiation to measure the dose profile induced by a microbeam array. They demonstrated the feasibility of real-time measurements and a consistent detector response for a dose rate between 1 and 700 Gy s^−1^ (Livingstone *et al.*, 2016 ▸). Using electrons in FLASH-irradiation conditions, Kranzer *et al.* (2022 ▸) studied the response of customized-readout microdiamonds. They concluded that the main source of response saturation under ultra-high dose-per-pulse conditions (up to 6.5 Gy and 2.5 µs pulse duration) was due to readout circuit impedance rather than charge recombination (Kranzer *et al.*, 2022 ▸). However, these sub-millimetre-sized dosimeters only provide a punctual response.

The following work aimed to develop and characterize a prototype striped diamond portal detector for online treatment monitoring during synchrotron MRT treatments. The work is divided into three steps. First, laboratory experiments combined with synchrotron irradiation tests performed on a bulk detector allowed us to check the effectiveness of monocrystalline diamonds to monitor high-photon-flux measurements. Subsequently, after an initial phase of Monte Carlo simulations to characterize the optimal detector thickness, a striped detector was tested to simultaneously control a few microbeams at the center of the field. Finally, the last step consisted of comparing the portal monitoring of the transmitted microbeams through phantoms irradiated under MRT conditions with Monte Carlo simulations.

## 2. Materials and methods

### 2.1. Synchrotron radiation

Experiments were conducted at the ID17 biomedical beamline of the European Synchrotron Radiation Facility (ESRF, Grenoble, France). The beamline is made up of two hutches: the ‘MRT hutch’ and the ‘monochromatic hutch’. The MRT hutch focuses on technique development, while the monochromatic hutch allows the use of almost monochromatic photon beams. The MRT hutch, situated around 40 m from the source, employs a wiggler insertion device. Generated photon flux travels through filters and collimators within a vacuum section to shape the beam and remove lower-energy components.

The beam structure is composed of 200 to 1000 benches, spanning a distance of just under 900 m (the circumference of the ring). Therefore, it can be considered to provide a quasi-continuous beam on a <50 ns scale. After filtration (beryllium: 2.3 mm; carbon: 1.13 mm; aluminium: 1.95 mm; copper: 2.08 mm; aluminium: 2 mm; gold: 0.3 µm), a photon spectrum extending from 50 to 500 keV (average energy: 121 keV) is produced (Pellicioli *et al.*, 2021 ▸; Adam *et al.*, 2022 ▸). In this configuration the dose rate is 5000 Gy s^−1^. The produced beam has a horizontal divergence of the order of 1 mrad and a vertical divergence of the order of 1 µrad.

A multi-slit collimator (MSC) transforms the homogeneous field generated by the synchrotron source into a spatially fractionated array of microbeams (Bräuer-Krisch *et al.*, 2009 ▸). The MSC used for this study is 8 mm thick (along the beam propagation direction) and is realized by assembling 125 plates made out of tungsten carbide alloy (Pellicioli *et al.*, 2021 ▸). The plates are machined and assembled side by side with micrometric precision in a copper frame to create 50 µm-wide and 3 mm-high openings, separated by 400 µm.

In contrast, the monochromatic hutch is positioned 150 m from the light source. Photons reach the MRT setup and additional tubing maintains photon propagation to the experimental arrangement. A monochromator, composed of two (111) silicon crystals in Laue geometry, allows a given energy to be selected (25–130 keV range) with a small bandwidth (Δ*E*/*E* ≃ 10^−3^), and dose rates from 0.1 to 1 Gy s^−1^.

### 2.2. Monocrystalline diamond detectors: description and first characterization

**Figure 1:**
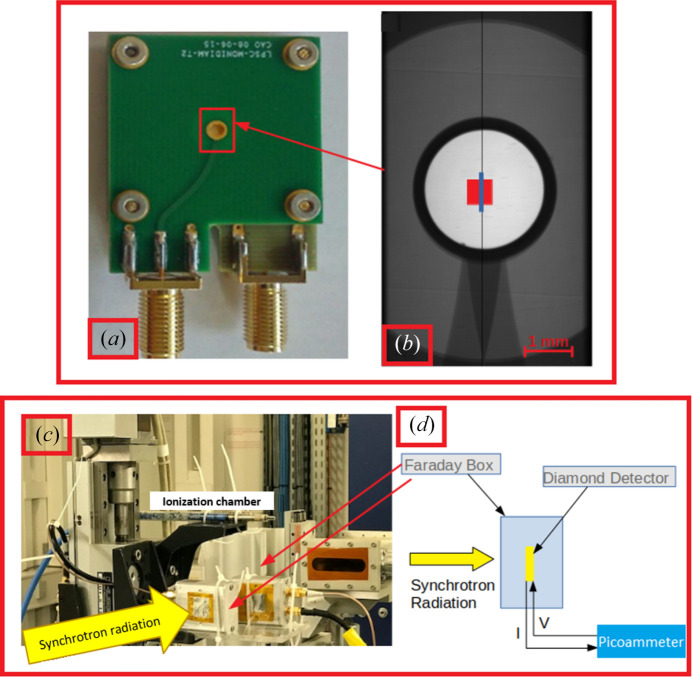
(*a*) Detector mounted on a printed circuit board (PCB) holder and (*b*) corresponding detector radiography (23 µm × 23 µm pixel size): in red the beam spot (0.5 mm × 0.5 mm) and in blue one microbeam (50 µm × 795 µm) are graphically painted. (*c*) Image of the experimental set-up used to test the diamond detector under synchrotron radiation for energy and dose-rate characterization. (*d*) Schematic representation of the experiment.

Two monocrystalline diamond detectors [4.5 mm × 4.5 mm surface area, Figs. 1 ▸(*a*) and 1(*b*)] obtained by chemical vapor deposition were analyzed: one of thickness 550 µm having a TiAl metallization, and one of thickness 150 µm having an Al metallization. Notably, the diamond detectors employed in this work originate from commercial diamonds provided by Element Six Ltd (UK) and their specific characteristics can be found online (Element Six, 2020 ▸).

Both detectors were placed between two printed circuit boards to facilitate assembly/disassembly of the samples. For the bulk diamond characterization, laboratory tests were conducted at our institution. First, the leakage current at different bias voltages was measured to check the quality of both the electronics and the metallization. Then, diamonds were tested under α-particle irradiation (^241^Am source, 5.5 MeV mean energy) using two techniques: a transient current technique to characterize the displacement of the charges, and spectroscopy to quantify the charge collection efficiency. Irradiations under a conventional X-ray tube (0.5 mm × 0.5 mm photon beam, 18 mA, 160 kV energy, ∼1 Gy min^−1^ dose rate) were performed to measure the current generated by the charge displacement at low dose rate. Each diamond was placed in an aluminium box for electromagnetic protection, and a circular opening covered with a 12 µm-thick layer of aluminized Mylar was used to allow passage of the beam. Subsequently, measurements under synchrotron radiation were performed (i) to determine the energetic characterization of the diamonds, and (ii) to study the diamonds’ response as a function of dose rate in a microbeam [Figs. 1 ▸(*c*) and 1(*d*)]. For case (i), a 0.5 mm × 0.5 mm monochromatic beam (33–130 keV energy range) was employed in the ID17 monochromatic hutch building, and an ionization chamber was used as a reference to address any dose-rate variations (between 0.39 and 0.85 Gy s^−1^). A Keithley 6487 picoammeter was used to measure the current (20 ms integration time) and as a voltage source (−500 V to the thickest diamond and −150 V to the thinner diamond). Irradiations (5 × 20 s) were performed for each energy, and experimental results were compared with the theoretical value obtained from

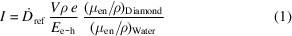

where 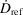 corresponds to the reference dose-rate in water for the same irradiation field, *V* is the irradiated volume, ρ the density of diamond (3.52 g cm^−3^), 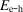 the average electron–hole creation energy [13.1 eV in diamond (Pan & Kania, 1994 ▸; Gaowei *et al.*, 2015 ▸)] and 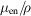 the mass absorption coefficient.

Maintaining the same readout setup [Figs. 1 ▸(*c*) and 1(*d*)] in case (ii), working in the MRT hutch, a polychromatic microbeam (50 µm × 795 µm collimation) was used for the irradiations with the beam intensity being tuned using various PMMA absorbers. The reference dose-rate was measured with a PTW PinPoint ionization chamber (PTW, Freiburg, Germany) at 2 cm depth in solid water using a 20 mm × 20 mm irradiation field (Fournier *et al.*, 2016 ▸).

### 2.3. Monte Carlo simulations

Monte Carlo simulations were performed with *GATE* (*Geant4 Application for Tomographic Emission*) (Sarrut *et al.*, 2014 ▸) using the Livermore-polar physics list to account for physical processes at low energy (<600 keV) and photons’ polarization due to synchrotron radiation (100% polarization in the horizontal plane). The primary slit and the multileaf collimator were considered perfect, not allowing the passage of photons through the material. Simulations were performed for different diamond thicknesses, between 50 and 550 µm, using nine microbeams (source-to-detector distance = 47.3 m, collimator-to-detector distance = 6.6 m, irradiated detector surface width/height = 58 µm/924 µm) to irradiate the entire diamond chip in width. One billion particles were simulated in the appropriate spectrum and for energies <600 keV.

### 2.4. Striped diamond detector

**Figure 2:**
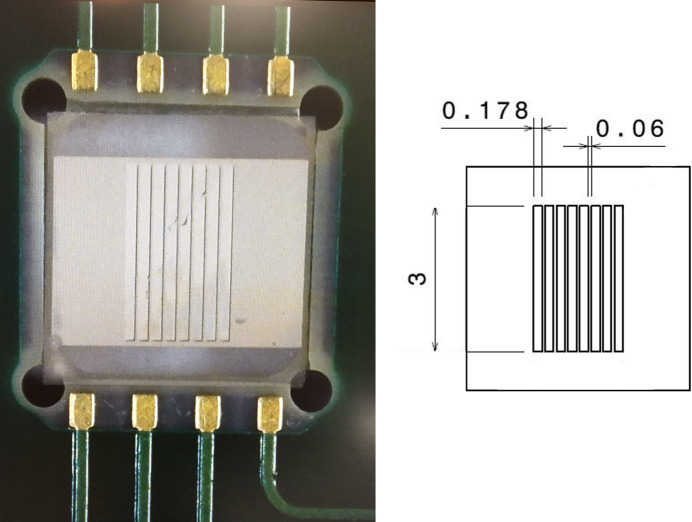
Image of the prototype of the eight-strip diamond (left) with no guard ring (the back-side metallization is visible by transparency). On the right, a schematic representation where dimensions are in millimetres.

Starting from a 550 µm-thick monocrystalline diamond, an eight-strip (each strip 3 mm high, 178 µm wide and 60 µm equally spaced) detector was developed (Fig. 2 ▸). The back side of the diamond was fully metalized across an area of 3.2 mm × 3.2 mm, and on the front side a thin layer of aluminium was deposited as strip metallization. In this way, linear electric field lines (typically 1 V µm^−1^) are induced between the strips and the reverse of the diamond, allowing charge collection when applying a bias voltage (typically applied on the fully metallized side). The metal coating was performed at the Nanofab laboratory.

The dimensions of the strips were selected based on size, spacing and horizontal divergence of the synchrotron’s microbeams, allowing visualization of alternating microbeam and interbeam areas. To enable simultaneous reading from the strips, an electronic system based on a charge-to-digital converter (QDC) was developed with an integration time between 1 and 100 ms (Gallin-Martel *et al.*, 2016 ▸). Furthermore, an analog-to-digital converter (ADC) was introduced to account for residual charges, which are not measured by the QDC. In the final detector prototype, a guard ring surrounding all strips was added to prevent charges generated outside the active zone from being collected by the strips and to keep the uniformity of the electric field up to the extreme strips. Finally, detector characterization was carried out under synchrotron irradiation by performing a horizontal scan with one microbeam (25 µm steps).

### 2.5. Phantom tests

**Figure 3:**
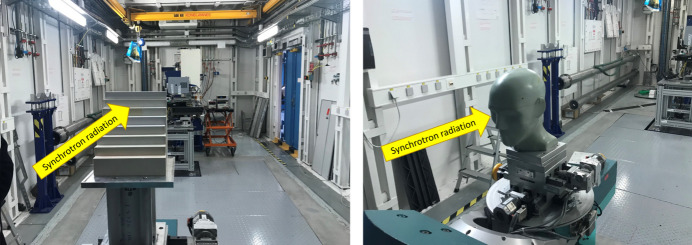
Photographs of the RW3 staircase phantom with thicknesses ranging from 2 to 16 cm (left) and of the human head phantom (right). The detector was positioned 6 m behind the phantoms.

First, MRT measurements were made using a staircase RW3 water equivalent (density 1.05 g cm^−3^) homogeneous phantom (Fig. 3 ▸, left). Then, experimental results were compared with Monte Carlo simulations, using 5 × 10^9^ photons per RW3 thickness. The beam was shaped to obtain five microbeams on the detector plane. During the irradiation, a vertical scan of the phantom (constant speed of 5 mm s^−1^) was performed to create the desired vertical field size. The detector integration time was set at 10 ms. For each thickness the measured values were averaged, and the uncertainty was determined as the standard deviation of the measured values.

Irradiations were also conducted on a realistic CIRS human head phantom (Fig. 3 ▸, right) to test the detector’s response in the presence of a non-homogeneous object. A five microbeams irradiation geometry was used. Then, Monte Carlo simulations were performed using a 3D tomographic image of the human head phantom implemented in *GATE*, which was divided into three materials: air, brain (1.04 g cm^−3^ density) and skull material. The geometry of the simulation was adapted such that the vertical dimension of one voxel (2.5 mm) matched the detector acquisition of 62.5 ms to account for the dynamic irradiation procedure at constant speed.

## 3. Results

### 3.1. Monte Carlo simulations: influence of detector thickness

**Figure 4:**
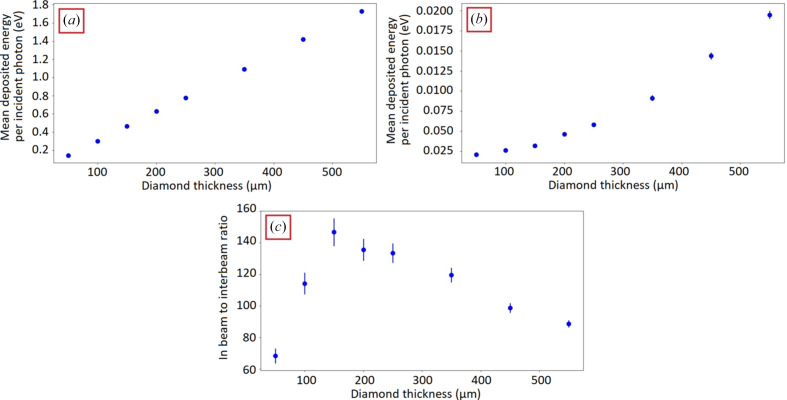
Calculated response of the diamond as a function of thickness in (*a*) a strip facing a microbeam and (*b*) a strip facing an interbeam. (*c*) Ratio of the average energy deposited in a strip facing a microbeam and in the adjacent strip facing an interbeam (averaged over the two adjacent strips). Calculated for energies <600 keV.

The variation of the detector response as a function of the diamond thickness was analyzed accounting separately for a strip facing a microbeam [Fig. 4 ▸(*a*)] and for a strip facing a valley [Fig. 4 ▸(*b*)]. Regarding the first case, the detector response was found to be proportional to the diamond thickness, whereas, for the second case, a linear behavior was not observed [Fig. 4 ▸(*b*)]. This was due to the non-linear variations in (i) the energy deposited by scattered photons and in (ii) the energy loss due to the secondary electrons escape phenomenon. In addition, the ratio between the response of the central strip and the average of the two adjacent strips was simulated for each thickness [Fig. 4 ▸(*c*)]. The maximum ratio was observed at 150 µm, resulting in the thickness value able to minimize the background noise in the interbeam areas.

### 3.2. Characterization of monocrystalline diamonds

**Figure 5:**
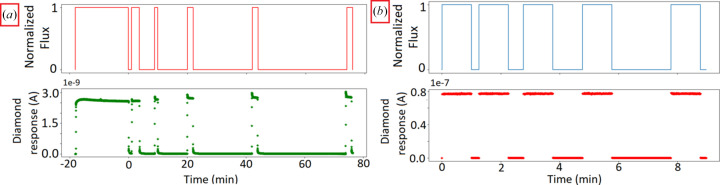
Response of the 550 µm diamond during 160 kV X-ray tube irradiation (*a*), and 95 keV monochromatic synchrotron radiation (*b*). During both irradiations the irradiated area was 0.5 mm × 0.5 mm.

The spectroscopy tests showed that the charge collection efficiency was approximately 100% (Gallin-Martel *et al.*, 2021 ▸). First irradiations were performed using the 550 µm-thick TiAl-covered diamond (irradiation area: 0.5 mm × 0.5 mm) under a 160 kV X-ray tube (1 Gy min^−1^ dose rate) for 20 min. First measurements were conducted during the heating phase of the tube, to allow stabilization of both photon flux and detector response. Indeed, the diamond exhibited a transient behavior: the signal increased reaching the maximum value after a few minutes (overshoot) and then slowly decreased [Fig. 5 ▸(*a*)]. After each irradiation pause, during which thermal de-trapping of hollow traps occurred, an over-response was observed with a fast decay of a few seconds before reaching the plateau. This plateau corresponded to a dynamic equilibrium between the trapping and de-trapping of both hollow and deep traps, resulting in an overall charge collection efficiency of 100% (Guerrero *et al.*, 2005 ▸). In addition, an increase in the over-response was observed by increasing the time without radiation [Fig. 5 ▸(*a*)].

The same test was performed under synchrotron radiation (95 keV monochromatic X-rays, 0.5 Gy s^−1^ dose rate, and irradiation pauses between 15 and 120 s), where the previously observed over-response disappeared [Fig. 5 ▸(*b*)].

**Figure 6:**
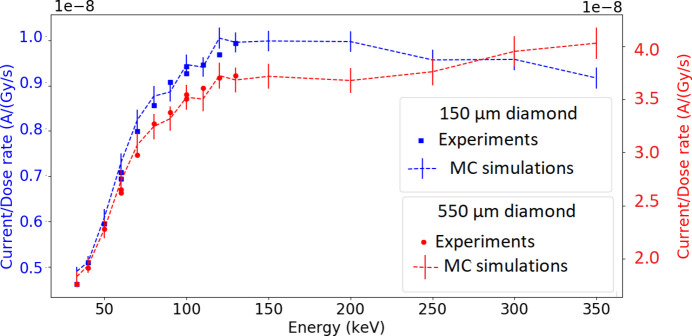
Dose-rate normalized current measurements of the diamonds in water as a function of energy, along with Monte Carlo (MC) simulations.

The energy-dependent dose-rate normalized current measurements were compared with Monte Carlo simulations (Fig. 6 ▸), showing an agreement within 5%. Two different behaviors depending on the diamond thickness were observed at energies higher than those used in our measurements: thicker diamonds showed an increase in simulated responses with increasing energy, while thinner diamonds showed a decrease in simulated responses with increasing energy.

**Figure 7:**
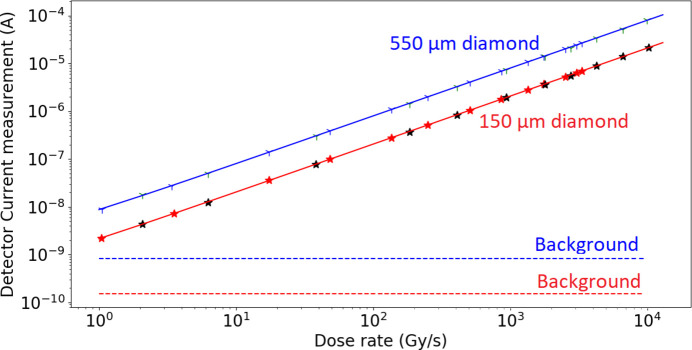
Responses of the two diamond detectors as a function of the dose rate measured at the MRT hutch, using a filtered polychromatic beam and various PMMA absorbers to reduce the dose rate. For each diamond thickness, two measurement sets were acquired. Horizontal dashed lines correspond to the background current level.

Finally, the responses of the two diamond detectors as a function of dose rate were studied (Fig. 7 ▸). To increase statistics, data were acquired during two different experiments, and leakage currents of 0.8 ± 0.3 nA and 0.15 ± 0.08 nA were detected for the thickest and the thinnest diamond, respectively. The detector’s current was found to be linear with the dose rate regardless of the thickness of the diamond, for dose rates ranging from 1 to 10^4^ Gy s^−1^.

### 3.3. Striped diamond detector

**Figure 8:**
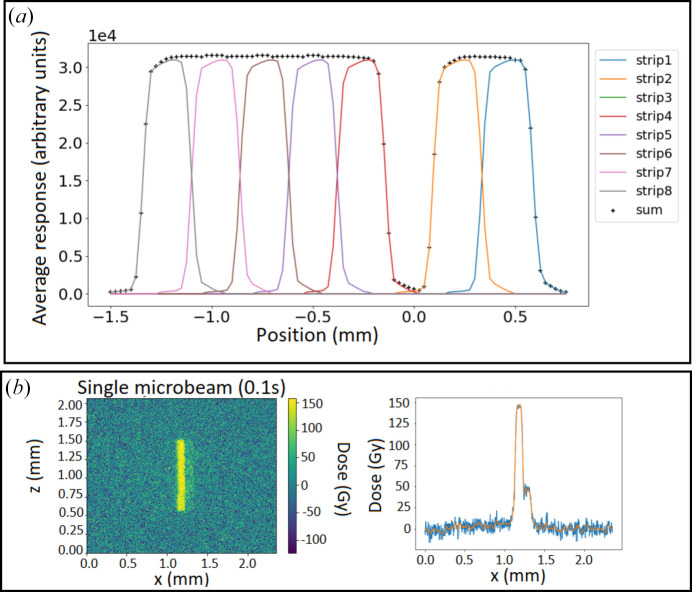
(*a*) Result of the horizontal scan with one microbeam. (*b*) Analysis of the Gafchromic film. On the left, the 2D transverse profile, and, on the right, the dose response along the horizontal axis within the microbeam area.

Figure 8 ▸(*a*) shows the responses of seven strips (the third one did not respond) of the 550 µm-thick diamond during a horizontal 25 µm-step scan using one microbeam. The average responses were found to be identical, and the sum of the signals was the same in all scanning positions for all strips, meaning that no electric charge was lost. Besides, the individual strip response was homogeneous; however, a reproducible asymmetry was observed for each strip. The lower signal on the left part of a strip was compensated by the appearance of a signal on the adjacent strip to the right, leaving the sum of the responses unchanged. In order to verify that the asymmetry was linked to the microbeam intensity, a radiochromic film irradiation was performed [Fig. 8 ▸(*b*)]. A Gafchromic HDV2 film positioned on the detector box was irradiated for 0.1 s under the same conditions as the detector. Results are shown in Fig. 8 ▸(*b*), where an asymmetry of the beam shape is visible, together with a secondary peak on the right-hand side whose value was one-third of the main peak.

Additionally, a full horizontal scan of the strips by a three-microbeam array was performed, and the results confirmed both the reproducibility of the strips’ response and the asymmetry of the microbeams produced behind the multi-slit collimator.

### 3.4. Phantom tests

**Figure 9:**
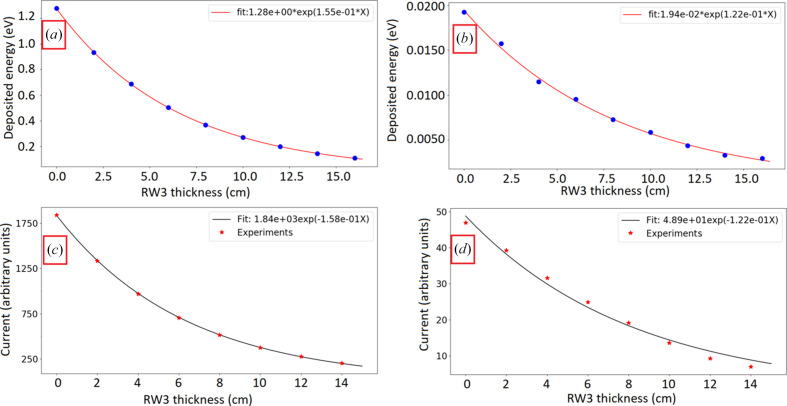
Detector response as a function of increasing RW3 thickness for the central strip facing a microbeam [(*a*) simulations; (*c*) experimental results], and for an adjacent strip facing an interbeam area [(*b*) simulations; (*d*) experimental results]. For the simulations, the deposited energy corresponds to the mean value per incident photon.

Simulations of the detector response for a strip facing a single microbeam and for a strip facing an interbeam area as a function of the traversed thickness of the step-shaped phantom are shown in Figs. 9 ▸(*a*) and 9(*b*). For a strip facing a microbeam, an exponential decay coefficient of 1.55 × 10^−1^ ± 0.01 cm^−1^ was obtained [Fig. 9 ▸(*a*)], resulting in a 3.7% difference from the National Institute of Standards and Technology (NIST) theoretical value (1.61 × 10^−1^ cm^−1^) for an average energy of 123.7 keV, corresponding to the mean photon energy in a zone facing a microbeam. For interbeam regions, a decay coefficient of 1.22 × 10^−1^ ± 0.04 cm^−1^ was obtained [Fig. 9 ▸(*b*)], differing by 2.5% from the theoretical value (1.19 × 10^−1^ cm^−1^) obtained for a mean energy value of the transmitted beam behind 8 mm of tungsten of 308 keV in a zone facing an interbeam region. Additionally, measurements were performed on the staircase RW3 phantom for strips facing a microbeam [Fig. 9 ▸(*c*)] and for strips facing an interbeam area [Fig. 9 ▸(*d*)]. Differences between experiments and simulations were lower than 2%.

**Figure 10:**
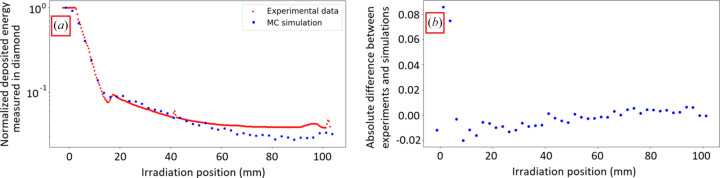
(*a*) Simulated (blue points) and measured (red stars) response of a diamond detector strip facing a microbeam during the scan of the anthropomorphic human head phantom. (*b*) Absolute differences between the experiment and the simulations.

Figure 10 ▸ shows the results of the anthropomorphic phantom scans by both simulation and experiment. Both curves [Fig. 10 ▸(*a*)] have a similar trend. In addition, the absolute difference between experiments and simulations was investigated, showing differences of <2% of the direct beam energy deposit [Fig. 10 ▸(*b*)]; except for the first two points which showed a larger absolute difference (around 8%) possibly due to the difference in pitch between the images (0.4 mm for the experiments versus 2.5 mm for the simulations).

## 4. Discussion

Our study aimed to develop and characterize a prototype of a striped diamond portal detector for online microbeam monitoring during synchrotron MRT treatments. The initial results obtained using an X-ray tube showed a significant variation in the detector’s response. During the tube heating phase, the detector response exhibited an undershoot caused by its stabilization. This phenomenon, also known as priming, occurs when deep traps in the diamond material become filled, contributing to an increase in the diamond’s charge collection efficiency and thus improving the stability of the detector (Guerrero *et al.*, 2005 ▸). After the pre-heating phase, at each beginning of an irradiation phase, the detector response presented an overshoot at least 10% higher than the measured current once stability was reached. This transient effect at the beginning of the irradiation is linked to the competition between the trap filling due to the particle flux and the un-trapping linked to the time without irradiation (Bergonzo *et al.*, 2007 ▸). However, in the present situation involving high dose-rates, the over-response was observed during the first cGy of dose deposition in the diamond at 1 Gy min^−1^ [visible in Fig. 5 ▸(*a*)]. This is completely smeared out in the first integration bin of the picoammeter, since the dose rate is close to 10^4^ Gy s^−1^ in the microbeams and even above 100 Gy s^−1^ in the interbeam areas. Therefore, these transient effects can be neglected in MRT where the irradiation time is of the order of a second, and the detector is fixed with respect to the beam. This confirms the possibility of using the diamond for dose monitoring during MRT irradiations.

The entire experimental study was conducted for two different diamond thicknesses: 550 µm and 150 µm. However, Monte Carlo simulations were performed to study the influence of the detector thickness on their response, showing that the optimal detector thickness to minimize the background noise in the interbeam areas was 150 µm. In this paper the results were presented for an eight-strip detector, designed on one single bulk diamond crystal. Future short-term projects include the realization of a first full-size detector prototype (to cover the whole MRT fields) to be assembled and tested in our laboratory and then characterized under synchrotron radiation. The diamond detector will be made up of an array of nine 150 µm-thick diamonds (17 strips each) with the same lateral size, to cover a 30 mm-wide irradiation field. Indeed, the whole treatment field will be covered using 153 strips allowing simultaneous measurements of 75 microbeams and 75 interbeams area. A detailed technical description of this multidetector and its electronics will be given in a forthcoming paper.

The behavior of both 150 µm- and 550 µm-thick diamonds as a function of dose rate was studied, showing a linear current response with increasing dose rate. This result is essential for the development of a portal dosimeter for fluence monitoring in MRT. Indeed, the use in portal mode and the possibility of tracking microbeams and interbeam areas imply a linearity of the detector’s response over four orders of magnitude, which has been demonstrated here. Previously, under synchrotron radiation, Livingstone *et al.* (2016 ▸) showed very good linearity of a PTW microdiamond allowing a point measurement up to a dose rate of 10^3^ Gy s^−1^. In the present study, we were able to confirm this linearity up to a higher dose-rate in water (1.2 × 10^4^ Gy s^−1^), well above the nominal dose-rate used in MRT (6 × 10^3^ Gy s^−1^). In addition, diamonds used in our study had a surface area of 4.5 mm × 4.5 mm making possible the measurement of several microbeams simultaneously, which is not possible with the PTW microdiamond. Note that the achieved response-linearity at dose rates up to 12 × 10^3^ Gy s^−1^ confirms results obtained at higher FLASH-irradiation rates with electrons, up to several MGy s^−1^. In this regimen, the intrinsic limitations of Schottky-diode diamonds are due to their electronic readout, and not to charge-carrier recombination in the material (Kranzer *et al.*, 2022 ▸).

Maintaining the same irradiation conditions, the diamonds’ energy characterization was performed, and an increase in the diamonds’ response was observed with increasing energy. The two diamond detectors showed a similar response as a function of energy with a growth of 0.3% per keV from an energy of 90 keV. This result implied that, in the context of MRT where a polychromatic beam is used, a correction factor depending on the energy of the beam transmitted through the patient would be applied to perform dosimetric measurements. It has been demonstrated by Livingstone *et al.* (2016 ▸) that this calibration factor could be chosen as the one measured for the monochromatic energy corresponding to the average energy of the MRT spectrum. It has also been shown that microdiamond detectors require radiation quality correction factors in order to be used in proton minibeam reference dosimetry (Ortiz *et al.*, 2022 ▸). In addition, for both detectors, we presented only comparisons between normalized data and calculations. Actually, both results are in overall agreement within the systematic uncertainties. One of the main uncertainties comes from the beam divergence, irradiating an active surface greater than 0.5 mm × 0.5 mm, as verified with Gafchromic film.

Furthermore, Monte Carlo simulations were performed to extend the diamonds’ energy characterization beyond 130 keV, and the results were in agreement with experimental values (within 5%). However, the two diamonds showed a different behavior depending on their thickness, related to a progressive establishment of electronic equilibrium as a function of material thickness, requiring a greater thickness at high energies.

After an initial testing phase, a transition from single-pixel diamond to a striped detector was made, enabling multiple microbeams to be monitored simultaneously. We demonstrated that there is no loss of charge collection efficiency, although the interstrip width (60 µm) is larger than the microbeam width, thanks to the high bias applied. First simulations performed to analyze the detector response as a function of the traversed thickness showed a good agreement with respect to the theoretical attenuation value both for the strips facing the microbeams and those facing the interbeam areas.

Finally, a first proof of the feasibility of monitoring a scan of an anthropomorphic head phantom under conditions typical of MRT treatment (scan speed of 40 mm s^−1^) showed the feasibility of monitoring a complete irradiation of a microbeam in real time. The average absolute differences between the normalized simulation and normalized experimental data was found to be lower than 2%.

So far, various methods have been studied to perform experimental dosimetry during synchrotron spatially fractionated irradiations (Bartzsch *et al.*, 2020 ▸; Bräuer-Krisch *et al.*, 2015 ▸; McErlean *et al.*, 2016 ▸; Siegbahn *et al.*, 2009 ▸). Currently, the state-of-the-art method for dosimetry in MRT involves measuring the dose in a reference broadbeam (2 cm × 2 cm) using a PinPoint ionization chamber (Prezado *et al.*, 2011*a* ▸; Crosbie *et al.*, 2013 ▸; Fournier *et al.*, 2016 ▸), and radiochromic films to determine peak and valley doses and their ratio (Ocadiz *et al.*, 2019 ▸; Pellicioli *et al.*, 2019 ▸; Day *et al.*, 2020 ▸). However, ionization chambers lack the spatial resolution required to resolve microbeam (or minibeam) peaks. Actually, Prezado *et al.* (2011*b* ▸) proposed an absolute dosimetry protocol for spatially fractionated synchrotron beams using dose-rate measurements in broad beams with a PinPoint chamber and conversion into minibeam peak doses using Monte Carlo calculations. Livingstone *et al.* (2016 ▸) have shown that microdiamond detectors can be a good alternative for experimental dosimetry in MRT; however, providing point measurements and long scanning times for retrieving even one-dimensional data. Overall, each of the aforementioned detector solutions lacks one or more of the essential characteristics required for online beam monitoring or real-time *in vivo* dosimetry protocols in spatially fractionated synchrotron radiotherapy.

In this work, we demonstrated that striped monocrystalline diamond detectors could provide a linear response as a function of the dose rate, a high spatial resolution, real-time monitoring and a reproducible response during synchrotron MRT treatments. These results clearly show the high potential of these detectors for MRT beam monitoring and real-time portal dosimetry.

## 5. Conclusions

Our results showed the first proof of feasibility of online monitoring during MRT treatments at synchrotron facilities. The tested diamonds showed a linear response as a function of dose rate, up to clinical rates of 1.2 × 10^3^ Gy s^−1^. A first prototype of a striped diamond portal detector was developed, showing to be efficient in performing individual measurements of each microbeam and valley areas. Online dosimetric measurements are currently ongoing during clinical veterinary trials at ESRF, and the next 153-strip detector prototype, covering the whole irradiation field, is being finalized at our institution and will be tested in the next months.
